# Cytotoxic and Immunomodulatory Effects of *Phormidesmis molle* Extract on Human Cells In Vitro

**DOI:** 10.3390/ijms27052236

**Published:** 2026-02-27

**Authors:** Ivanka Teneva, Krum Bardarov, Tsvetelina Batsalova, Dzhemal Moten, Balik Dzhambazov

**Affiliations:** 1 Faculty of Biology, Plovdiv University “Paisii Hilendarski”, 4000 Plovdiv, Bulgaria; teneva@uni-plovdiv.bg (I.T.); tsvetelina@uni-plovdiv.bg (T.B.); moten@uni-plovdiv.bg (D.M.); 2 InoBioTech Ltd., 1113 Sofia, Bulgaria; krum.bardarov@gmail.com

**Keywords:** cyanobacteria, *Phormidesmis molle*, extract, mass spectrometry, cytotoxicity, anticancer activity, ADMET, immunophenotyping, immunomodulatory properties

## Abstract

Cyanobacteria of the genus *Phormidesmis* are recognized as a promising source of biologically active secondary metabolites with anticancer and immunomodulatory properties. In the present study, we investigated both the cytotoxic and immunological effects of an extract obtained from *Phormidesmis molle* PACC (Plovdiv Algal Culture Collection) 8140 as well as its chemical composition. The extract was profiled by LC-ESI-MS/MS (Liquid chromatography—electrospray ionization—tandem mass spectrometry), and selected compounds were evaluated with in silico ADMET (Absorption, distribution, metabolism, excretion and toxicity) modeling. The cytotoxic potential of the extract was evaluated in vitro using the MTT (3-(4,5-dimethylthiazol-2-yl)-2,5-diphenyl tetrazolium bromide) assay on human colorectal adenocarcinoma cell lines (Caco-2, HT-29, and LS-180). The immunological impact of the extract was assessed on human peripheral blood mononuclear cells (PBMCs) isolated from healthy donors. PBMCs were treated with 100 µg/mL extract for 48 h, followed by flow cytometric immunophenotyping and ELISA (Enzyme-linked immunosorbent assay)-based cytokine quantification. The extract induced a concentration- and time-dependent decrease in cancer cell viability after 24, 48, and 72 h of exposure. At 72 h, treatment with the highest concentration (200 µg/mL) reduced cell viability to 74% in Caco-2 cells, 69–70% in HT-29 cells, and 59–61% in LS-180 cells. Morphological changes observed after treatment with *Phormidesmis* extract showed pronounced cytotoxic and apoptosis-related effects in the colorectal cancer cell lines tested. Immunophenotyping revealed a pronounced expansion of natural killer (NK) cells (CD56^+^ and/or CD16^+^). CD3^−^CD56^−^CD16^+^ NK population was markedly increased (from 67.7 ± 0.95% in non-treated PBMCs to 94.66 ± 0.90% in extract-treated PBMCs, *p* < 0.001). In contrast, the proportions of CD8^+^ T cells, CD19^+^ B cells, and CD11b^+^ monocytes were significantly reduced (from 21.5 ± 4.50% to 7.22 ± 0.41%, from 11.9 ± 1.70% to 6.06 ± 0.42%, and from 66.4 ± 0.60% to 34.4 ± 0.87%, respectively). Cytokine analysis demonstrated strong suppression of Th1-associated cytokines, with significantly reduced interferon gamma (IFN-γ, 461 ng/mL in controls vs. 84 ng/mL in extract-treated cultures) and tumor necrosis factor alpha (TNF-α) levels (169 ng/mL in controls vs. 32 ng/mL in extract-treated cultures), whereas nterleukin-6 (IL-6) was moderately elevated (from 158 ng/mL in controls to 234 ng/mL in extract-treated cultures) and IL-10 remained low. These findings demonstrate that *P. molle* extract combines cytotoxic activity against cancer cells with potent immunomodulatory effects, highlighting its potential as a source of bioactive compounds for immune-based therapeutic strategies.

## 1. Introduction

Cyanobacteria, including those of the genera *Phormidium* and *Phormidesmis*, produce a number of secondary metabolites. The phytochemical profile of these unique photosynthetic prokaryotes is particularly complex. It often contains unique metabolites not found in terrestrial plants. The ability of cyanobacteria to adapt to diverse environmental conditions, their long evolution, and cosmopolitan distribution likely led to the production of these diverse variants of secondary metabolites.

Species of the genus *Phormidium* and the genus *Phormidesmis* are less well studied than other genera of cyanobacteria (e.g., those producing cyanotoxins). However, studies spanning the period from the late 1990s to the present have gradually revealed the phytochemical potential of these genera. Cyanobacteria synthesize major classes of phytochemicals such as peptides and lipopeptides, alkaloids, polyketides and macrolides, phenols, glycolipids, and polysaccharides. These substances exhibit diverse biological activities ranging from cytotoxic and anticancer effects to antioxidant, anti-inflammatory, immunomodulatory, and antimicrobial properties. One of the most frequently reported biological effects of extracts from *Phormidium* and *Phormidesmis* species is cytotoxicity, especially against cancer cells. The first evidence for anticancer potential of these cyanobacteria comes from a comparative study by Teneva et al. [1]. Selective cytotoxicity was observed in one of the cyanobacterial samples, suggesting anticancer activity of the extract from this strain. Based on these data, Dzhambazov et al. [2] conducted a targeted study of two strains of *Phormidium molle* (now *Phormidesmis molle*). Extracts of *P. molle* showed dose-dependent cytotoxicity with pronounced effects on certain cancer cells and minimal effects on normal cells. Microscopic examination revealed that treatment with *P. molle* altered the cytoskeleton and disrupted the microtubule network of adherent cancer cells, leading to their rounding and disruption of the monolayer [2]. These findings demonstrate a clear anticancer effect in vitro, likely by interfering with the structural elements of the cell (microtubules) and inducing cell death in cancer cells, while sparing normal cells at equivalent doses. This selective cytotoxicity is a desirable feature for anticancer agents. Such activity is also exhibited by portoamides A/B, identified as cyclic peptides with a novel structure. They have been shown to cause a collapse of the mitochondrial membrane potential [3]. Portoamides are one of the leading compounds for the development of anticancer drugs. Pigment proteins such as C-phycoerythrin (C-PE) also have anticancer activity. C-PE shows dose-dependent suppression of cancer cell viability and induced apoptosis. Its mechanism is suggested to involve activation of the intrinsic (mitochondrial) apoptosis pathway and cell cycle arrest in G0/G1 [4].

*Phormidium*/*Phormidesmis* metabolites exhibit antioxidant properties, which is not surprising considering that many cyanobacteria produce pigments, polyphenols, and carotenoids that counteract oxidative stress. A recent comprehensive study by Georgiev et al. [5] evaluated the antioxidant activity of *P. molle* extracts. The extracts, rich in polysaccharide complexes and secondary metabolites, exhibited strong antioxidant activity. They also demonstrated immunomodulatory effects. It is suggested that the combination of sulfated polysaccharides and related metabolites likely contributes to reducing oxidative stress and improving the immune response.

In addition to cytotoxic, antitumor, and antioxidant activities, extracts from *Phormidesmis molle* and other *Phormidium* species also exhibit anti-inflammatory effects. Inflammation is a critical factor in the progression of various diseases, including cancer, and the anti-inflammatory properties of these extracts may contribute to their overall therapeutic efficacy. The membrane glycolipids monogalactosyldiacylglycerol (MGDG) and digalactosyldiacylglycerol (DGDG), isolated from *Phormidium* sp. ETS-05, have been shown to have significant anti-inflammatory activity [6]. Such galactolipids likely act by modulating eicosanoid pathways or through membrane-mediated signaling involved in inflammation (galactolipids from other sources are known to inhibit 5-lipoxygenase or COX enzymes).

Exopolysaccharides (EPS) and glycolipids (MGDG and DGDG) isolated from *Phormidium/Phormidesmis* strains are potential candidates for the treatment of chronic inflammatory diseases. Their ability to reduce inflammation without exhibiting toxicity [6] suggests that they could be used in the treatment of local skin inflammations (e.g., psoriasis, eczema) or even in systemic therapies of arthritis or inflammatory bowel diseases.

The antibacterial effects of *Phormidium papyraceum* extract are presented in a study by Teneva et al. [7], where a non-polar fraction was tested against a panel of bacteria. The extract showed broad-spectrum antibacterial activity, inhibiting both Gram-negative and Gram-positive bacteria. Chemical analysis of the extract by LC-MS/MS revealed several known bioactive compounds that likely contribute to the antibacterial effect: divertonol (a naphthalenone with antibiotic properties), torularhodin (a carotenoid pigment with antimicrobial activity), tanicolide (a polyketide lactone known from marine cyanobacteria), oligomycin C (a macrolide antibiotic) and even azithromycin (a well-known antibiotic, likely accumulated or produced by symbiotic microbes). The presence of these compounds in the *Phormidium papyraceum* extract suggests that either the cyanobacterium itself or its accompanying microbiome produces a cocktail of antimicrobial agents [7]. Overall, this extract exhibits multifaceted bioactivity, combining direct antimicrobial effects with immune system modulation—a desirable feature for anti-infective therapies.

Members of the genus *Phormidium* have been shown to produce neuroactive compounds with therapeutic value (neuroprotective and anti-Alzheimer’s agents). For example, *Phormidium retzii* has been shown to produce significant amounts of the plant sterol stigmasterol. Pure stigmasterol (0.45 μM) achieved 81% inhibition of acetylcholinesterase (AchE), with an IC_50_ of 0.214 μM, indicating high potency comparable to commercial anti-Alzheimer’s drugs [8]. Molecular studies have shown that stigmasterol binds to the active site of AChE, and its efficacy, accompanied by fewer side effects, makes it a promising agent for the therapy of Alzheimer’s disease [8].

In summary, *Phormidium* species, including *Phormidesmis molle* (syn. *Phormidium molle*), are rich sources of phytochemicals. The bioactive compounds found in *Phormidesmis* may have applications in multiple fields, including medicine and biotechnology, but more focused research on this genus is needed to fully understand the range of their properties. Although research on *Phormidesmis* is limited compared to other better-known genera of cyanobacteria, the potential for future discoveries remains significant. Despite the growing data on the phytochemical composition and biological activity of members of the phylum Cyanobacteriota (Cyanobacteria), and in particular those of the genus *Phormidium/Phormidesmis*, there remains a significant gap in knowledge regarding the specific compounds responsible for these properties. The objective of this study was to identify potential bioactive compounds in the *Phormidesmis molle* PACC 8140 extract, to evaluate its cytotoxic effects on human colon cancer cell lines and immunomodulatory impact on human peripheral blood mononuclear cells ex vivo, as well as to elucidate possible mechanisms of action, which are essential for understanding the therapeutic potential of this species.

Future research should focus on isolating novel compounds, identifying their precise mechanisms of action (e.g., molecular targets in cancer or inflammation), optimizing production (through cultivation or biosynthetic engineering), and evaluating their efficacy and safety in animal models. We believe that cyanobacteria of the genus *Phormidium/Phormidesmis* can contribute to the development and application of pharmaceuticals in oncology, immunology, neurology, and infectious diseases.

## 2. Results

### 2.1. LC-ESI-MS/MS Analysis of Phormidesmis Fractions

Extracted non-polar and polar fractions were analyzed by liquid chromatography—electrospray ionization tandem mass spectrometry (LC-ESI-MS/MS) in both negative and positive ion modes. Compound identification was performed by matching observed *m*/*z* and MS/MS fragmentation with ChemSpider, MZ cloud, Metabolica or other mass list resources. In total, over 550 distinct molecular features were tentatively identified across the four fraction/mode combinations (188 in non-polar negative, 180 in non-polar positive, 81 in polar negative, and 126 in polar positive modes). Data are provided in the Supplementary Materials (Chemical_composition.xlsx).

These metabolites span a wide range of chemical classes (from lipids and peptides to alkaloids, terpenoids, and carbohydrates) highlighting the rich secondary metabolite profile of the *Phormidesmis* extract. Notably, many of the identified compounds are already known with their biological activities., The major components and predominant compound classes in each fraction were analyzed.

#### 2.1.1. Non-Polar Fraction (Negative Ion Mode)

The non-polar extract analyzed in negative ESI mode yielded a diverse array of mostly lipophilic and aromatic compounds. We found a number of aromatic benzofuran and benzamide derivatives (e.g., a 4-ethoxy-benzamide linked to a benzofuran moiety), which indicates the presence of complex polyaromatic metabolites. Long-chain aliphatic sulfonates were also prominent. For example, laurylsulfonic acid (dodecane-1-sulfonic acid) was identified as a major component unique to the *Phormidesmis* extract sample (it was absent in the controls). Such alkyl-sulfonic acids act as surfactant-like metabolites and are rarely reported from cyanobacteria, suggesting a potentially novel lipid-derived constituent in the extract. In addition, an iridoid glycoside was tentatively identified as (+)-geniposide, a known bioactive terpenoid glycoside. Importantly, this non-polar fraction also revealed evidence of peptide metabolites. A compound matching paltolide C (an anabaenopeptin-type cyclic hexapeptide) was detected with a high sample/control ratio.

Another compound that we found in this fraction was a sterol derivative (tentatively methyl trioxochol-3-en acid), which suggests the presence of steroidal metabolites or terpenoids. Several heterocyclic alkaloids were also present, including complex piperidine/piperazine derivatives. In summary, the non-polar/negative fraction contained a broad mixture of hydrophobic secondary metabolites (including aromatic polyketides, long-chain lipids, terpenoid glycosides, and cyclic peptides), many of which are candidates for biological activity (e.g., protease-inhibiting peptides and anti-inflammatory iridoids).

#### 2.1.2. Non-Polar Fraction (Positive Ion Mode)

Conducted analysis of the non-polar fraction in positive ESI mode likewise identified a wide variety of secondary metabolites, with some differences in ionization reflecting complementary detection of certain compounds. Overall, this mode confirmed many lipophilic and aromatic compounds similar to the negative mode, such as benzofuran derivatives and phenylpropanoid-type molecules. For example, we identified multiple flavonoid-like chalcones (e.g., dimethoxy- and hydroxy-dimethoxyphenyl chalcone structures), which suggest the presence of polyphenolic constituents. This fraction was also rich in terpenoids. For example, the lupane-type triterpenoid moronic acid (C_30_H_46_O_3_) was detected with high confidence. In addition to triterpenes, a sesquiterpene was prominently detected—nootkatone (C_15_H_22_O), a characteristic grapefruit sesquiterpenoid, which was observed in this fraction at high levels.

The non-polar/positive fraction also contained several nitrogenous metabolites. Additionally, we found a thioadenosine derivative identified as 5′-S-(2-aminoethyl)-5′-thioadenosine, which points to sulfur-containing purine metabolites (potentially arising from S-adenosylmethionine/thiopurine pathways). Other compounds included phthalate esters and bis(4-ethylbenzylidene)sorbitol (a polymer additive), but these were present also in the control extracts. The predominant classes in the non-polar positive fraction were terpenoids, phenolic polyketides, and miscellaneous alkaloids. Many of these (e.g., moronic acid, nootkatone, chalcones) are known with their bioactivity, reinforcing that the lipophilic fraction of *Phormidesmis* harbors compounds with potential pharmaceutical or agrochemical interest.

#### 2.1.3. Polar Fraction (Negative Ion Mode)

The polar extract analyzed in negative mode was, as expected, enriched in more hydrophilic constituents, including complex lipids and polar metabolites. A key finding in this fraction was the identification of sulfonated glycolipids characteristic of cyanobacterial membranes. In particular, a sulfoquinovosyldiacylglycerol (SQDG) was detected, with a molecular composition consistent with a C_16:0_/C_18:2_ sulfoquinovosyl glyceride (palmitic and linoleic acyl chains on a sulfoquinovose headgroup). Overall, the most significant authentic components in this fraction appear to be glycolipids and other primary metabolites from the cyanobacterium’s cellular makeup.

#### 2.1.4. Polar Fraction (Positive Ion Mode)

The positive ESI analysis of the polar fraction revealed numerous highly polar, water-soluble metabolites, including sugars, amino acid derivatives, and other primary metabolites. Our analyses showed that oligosaccharides were especially abundant. For example, the pentasaccharide maltopentaose (a chain of five glucose units) was identified in this fraction at very high levels in the sample. This likely derives from partial hydrolysis of extracellular polysaccharides or storage glucans produced by the cyanobacterium. Another sugar-based metabolite that we detected was amygdalin, a disaccharide glycoside of mandelonitrile (famous as a plant cyanogenic glycoside). The tentative identification of amygdalin (or a structural isomer) in *Phormidesmis* is intriguing—it suggests the presence of complex glycosides in the polar extract, which might be microbial analogs of plant natural products. These sugar-containing compounds indicate that the polar fraction holds not only primary metabolites, but also secondary metabolites conjugated to sugars (which can enhance solubility and bioactivity). Additionally, the Maillard-type sugar derivative 3-deoxyglucosone (a reactive carbonyl sugar formed from glucose dehydration) was found, possibly as a byproduct of sugar metabolism or sample processing.

Importantly, the polar fraction was rich in nitrogenous osmolytes and antioxidants. We observed a prominent signal for *L*-ergothioneine, a rare sulfur-containing amino acid derivative (thiourea betaine of histidine). We also identified other small N-heterocycles, including dihydropyrazine derivatives (e.g., 2-butyl-3,5-dimethyl-5,6-dihydropyrazine) and octahydro-pyridopyrazine. These could be cyclic dipeptides or Strecker degradation products from amino acids; such compounds have been reported from microbial cultures as flavor or signaling molecules. A sesquiterpenoid was also detected in this polar run (nootkatone, as noted earlier, though in this run it appeared at low levels, possibly due to cross-sample carryover). Finally, it is worth noting that many highly polar metabolites (e.g., amino sugars, nucleosides, small organic acids) likely fall under the detection in this mode—indeed, the presence of maltose derivatives and amino acid betaines indicates a suite of primary metabolites supporting the physiology of the cyanobacterium.

### 2.2. Cytotoxicity Against Human Colon Cancer Cell Lines

**Figure 1:**
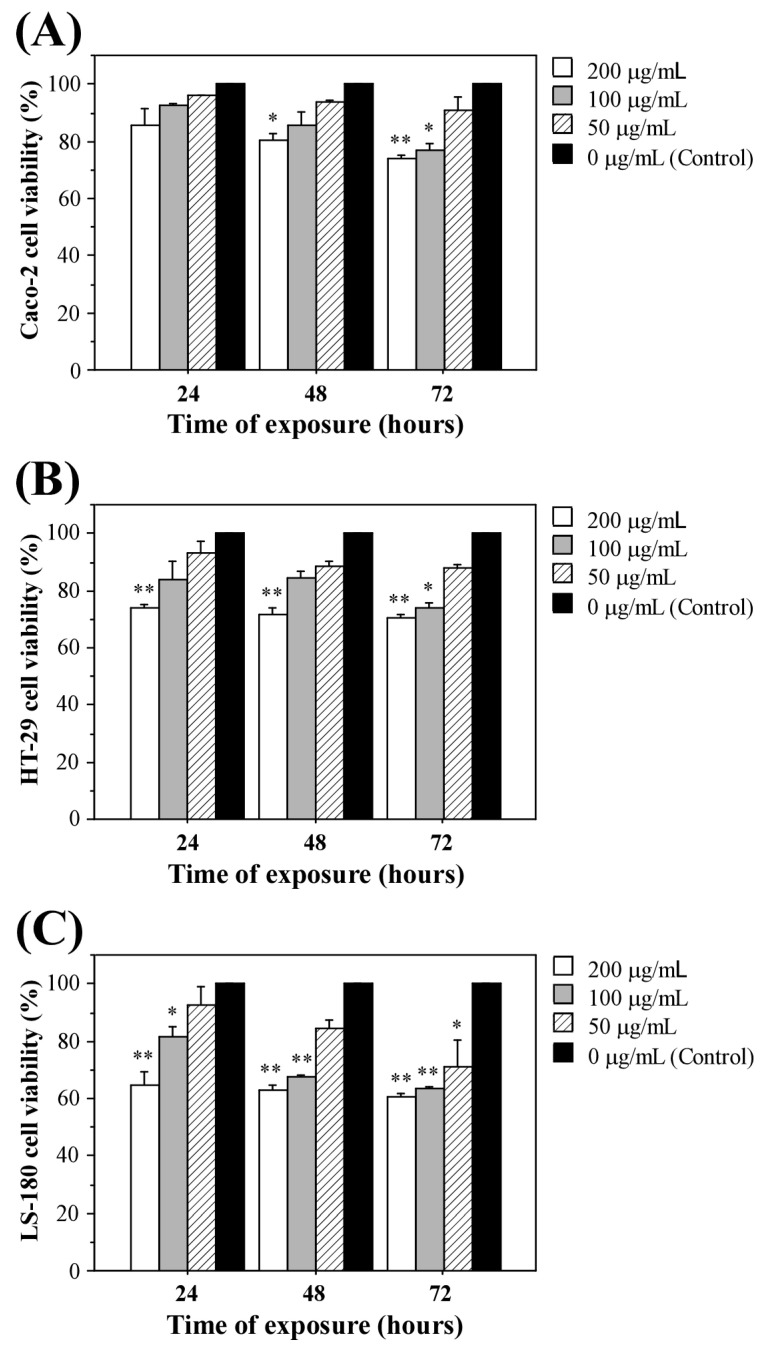
Effects of the non-polar fraction of *P. molle* extract on the viability of human colorectal adenocarcinoma cell lines evaluated by the MTT assay. Panels show cell survival of Caco-2 (**A**), HT-29 (**B**), and LS-180 (**C**) cells after exposure to increasing concentrations of the *P. molle* extract (50, 100, and 200 µg/mL) for 24, 48, and 72 h. Non-treated cells receiving equal amount of DMSO only (0 µg/mL, control) were used to define 100% viability. Data are presented as mean ± SD from three independent replicates. Statistical differences between treated and control groups were assessed using the Mann–Whitney U test; * *p* < 0.05 and ** *p* < 0.01 indicate significant reductions in cell viability relative to the control.

The non-polar *P. molle* extract showed a clear, dose- and time-dependent cytotoxic effect on all three colon carcinoma cell lines (Figure 1). At 24 h, the lowest concentration (50 µg/mL) had little impact on the viability (Caco-2 and HT-29 cells remained 94–96% viable and 88–97% of LS-180 cells compared to the control). Higher doses (200 µg/mL) reduced viability at 24 h to 81–90% in Caco-2, 73–74% in HT-29, and 61–68% in LS-180 cells. As exposure time increased, viability decreased further for all lines. At 72 h, these values declined to 74% (Caco-2), 69–70% (HT-29), and 59–61% (LS-180) at 200 µg/mL. Even at an intermediate dose (100 µg/mL), viability at 72 h dropped to 75–78% in Caco-2, 72–79% in HT-29, and 63% in LS-180. Statistical analysis indicated that viability reductions were significant (vs. control) at higher concentrations and longer exposure time (200 µg/mL, 72 h).

All cell lines were sensitive to the extract, but with distinct response patterns. LS-180 cells were the most susceptible—even at 24 h and 200 µg/mL their viability fell to 60–64%, significantly lower than that of Caco-2 or HT-29 cells under the same conditions. This pattern persisted at later times. Thus, LS-180 cells consistently showed the greatest viability loss, followed by HT-29 and then Caco-2 cell line.

To quantify potency, dose–response curves were fit by non-linear regression to estimate IC_50_ values at 72 h. The resulting IC_50_ was 63 µg/mL in LS-180 cells, 71 µg/mL in Caco-2, and 94 µg/mL in HT-29. These values underscore the strong cytotoxic effect of the extract, especially against LS-180 cells, consistent with their greater viability loss in the assays. The robust, dose-dependent viability inhibition observed (with statistically significant reductions at higher concentrations) highlights the potential anticancer activity of non-polar fraction from *P. molle* extract.

**Figure 2:**
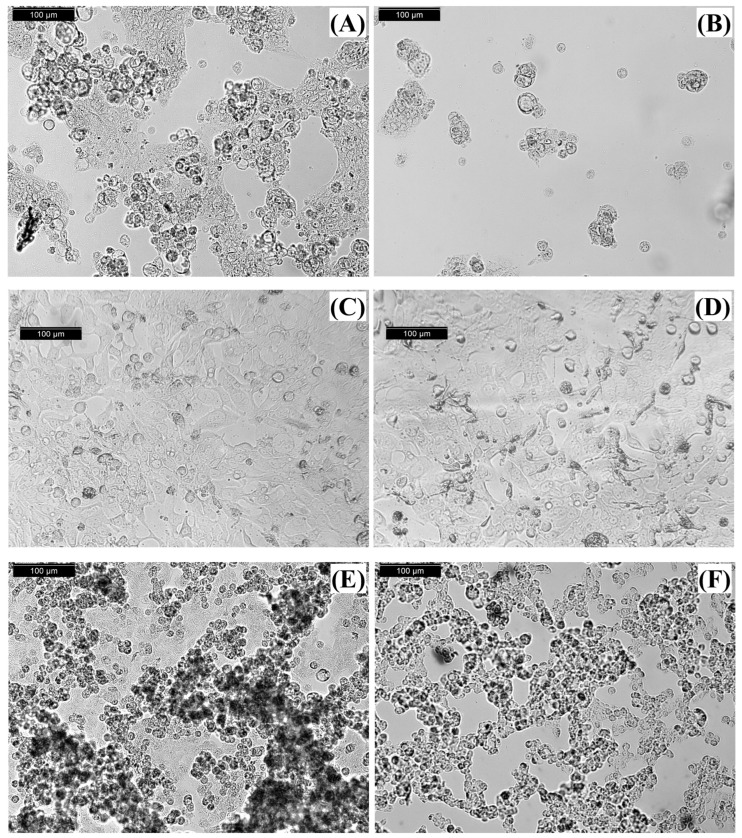
Effects of the *Phormidesmis* extract (200 µg/mL) on colorectal cancer cell lines 72 h after treatment. (**A**) non-treated Caco-2 cells, (**B**) extract-treated Caco-2 cells, (**C**) non-treated HT-29 cells, (**D**) extract-treated HT-29 cells, (**E**) non-treated LS-180 cells, (**F**) extract-treated LS-180 cells. Bar 100 µm.

To assess any possible morphological changes after treatment with the extract, photographs of the non-treated and treated cells were taken at the end of the experiment (Figure 2). In the non-treated control (Figure 2A), Caco-2 cells formed a dense, confluent epithelial monolayer with flat, polygonal morphology. After 72 h of exposure with 200 µg/mL extract (Figure 2B), cell density was markedly reduced and many cells took on a rounded, shrunken shape. Numerous treated cells appear detached from the substrate or floating. HT-29 controls (Figure 2C) also showed tightly packed epithelial cells and extensive cell–cell contacts. In the treated samples (Figure 2D), many HT-29 cells become visibly rounded and smaller, with reduced contact with neighboring cells. Non-treated LS-180 cells (Figure 2E) formed a confluent layer of epithelial cells. After extract treatment (Figure 2F), the culture density was lower, many LS-180 cells were rounded or irregular in shape and appear to have lifted off the surface. These rounded, detached cells leaved empty spaces in the monolayer.

### 2.3. ADMET Analysis of Selected Metabolites

The data presented were obtained by in silico pharmacokinetic profiling of the isolated compounds evaluating their absorption, distribution, metabolism, excretion and toxicity (ADMET) properties [9]. The ADMET profiles of our selected 22 compounds, presented in Table 1, were analyzed to determine which of them are most suitable for future drug development. The results are presented in the Supplementary Materials (Tables-ADMET.docx).

#### 2.3.1. Absorption and Distribution

The absorption and distribution profiles (Table S1) revealed considerable diversity among the 22 compounds. Most compounds showed moderate to good water solubility (between −2.9 and −6.8 log mol/L), with a general trend toward high intestinal absorption (>80% for the majority of compounds). Notably, compounds **2**, **5**, **13**, **15**, **16**, **19**, and **20** exhibited excellent intestinal absorption (>92%), suggesting strong potential for oral bioavailability. Caco-2 permeability values indicated that compounds **2**, **5**, **16**, **19**, and **20** had particularly favorable epithelial transport, consistent with efficient uptake across intestinal barriers.

Blood–brain barrier (BBB) permeability was limited for most molecules (negative logBB values), which is advantageous for compounds intended for peripheral pharmacological targets but may limit central nervous system (CNS) applications. Only compounds **2**, **5**, and **13** showed moderate CNS permeability, suggesting possible neuroactive potential. The fraction unbound (Fu) values indicated that several compounds, such as compound **17** (Fu = 0.457) and compound **22** (Fu = 0.427), remain more available in plasma compared to highly protein-bound molecules (e.g., compound **15**, Fu = 0.04). Overall, the data suggest that compounds **2**, **5**, **13**, **16**, and **19** represent the best candidates for systemic exposure due to high intestinal absorption and favorable permeability characteristics.

#### 2.3.2. Metabolism and Excretion

The metabolism and excretion profiles (Table S2) demonstrated that nearly all compounds are predicted to be CYP3A4 substrates, implicating potential metabolism by hepatic microsomal enzymes. A smaller subset (compounds **3**, **11**, and **18**) were predicted CYP2D6 substrates, while compound **3** and **11** also inhibit CYP2D6, raising the possibility of drug–drug interactions. Several compounds (e.g., compounds **1**, **3**, **12**, **19**) inhibited CYP2C19 or CYP2C9, suggesting variable risks for interference with major metabolic pathways.

Total clearance values showed marked differences. Compounds **10**, **14**, and **20** demonstrated the highest clearance rates (log clearance > 1.7), predicting rapid elimination and potentially reduced systemic half-life. In contrast, compounds **1**, **4**, **7**, **15**, **17**, **21**, and **22** had negative clearance values, indicating a propensity for slower elimination and possible accumulation with chronic dosing. Renal OCT2 transport involvement was minimal, with only compound **19** predicted as a substrate, which may influence its renal handling. From a pharmacological perspective, compounds **2**, **5**, **10**, **14**, and **20** strike a favorable balance of metabolic stability and clearance, while compound **19** requires closer attention due to its CYP inhibition profile and renal substrate status.

#### 2.3.3. Toxicity

The toxicity assessment (Table S3) highlighted a generally favorable safety profile for most compounds, though with some outliers. AMES mutagenicity was predicted only for compounds **3**, **11**, and **16**, raising potential genotoxicity concerns. hERG inhibition—an important predictor of cardiotoxicity—was generally absent, though hERG II inhibition signals were seen for several compounds (e.g., compounds **1**, **3**, **4**, **6**, **7**, **11**, **12**, **15**, **17**, **18**, and **22**), suggesting caution in cardiac safety evaluation.

Regarding systemic tolerance, compounds **5**, **14**, **17**, and **21** exhibited relatively higher maximum tolerated dose predictions, while compounds **2**, **6**, **9**, and **20** showed lower values, implying narrower safety margins. Hepatotoxicity predictions were positive for multiple compounds, including compounds **1**, **3**, **4**, **6**, **7**, **11**, **12**, **15**, **17**, **18**, and **22**, marking liver safety as a key consideration for these candidates. In contrast, compounds **2**, **5**, **9**, **14**, **19**, and **21** were consistently negative for hepatotoxicity risk. Fish and protozoan toxicity (Minnow and *Tetrahymena pyriformis* assays) were variable, but environmentally, compounds **14**, **17**, and **22** had notably high predicted aquatic toxicity, suggesting ecological impact concerns.

**Figure 3:**
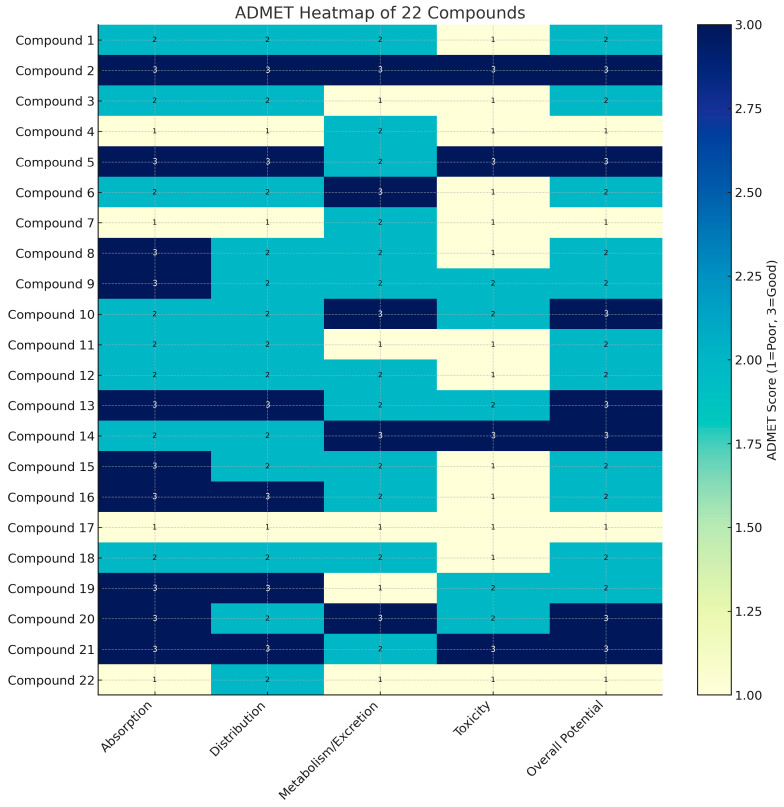
ADMET property heatmap for selected 22 compounds. Each row corresponds to a compound (**1**–**22**), and each column represents one ADMET criterion (absorption, distribution, metabolism/excretion, toxicity, and overall potential). Scores are categorical (1 = poor, 2 = moderate, 3 = good) and indicated by color intensity (darker shades denote more favorable properties).

We have prepared a heatmap of the ADMET profiles for the 22 analyzed compounds (Figure 3).

Each compound’s overall potential score (final column in Figure 3) provides an integrated view of its pharmacokinetic favorability. These scores span a scale from 1 (poor) to 3 (good), capturing the degree to which a compound meets key criteria for drug development. The heatmap reveals that only a handful of compounds approach the maximum overall score, while many receive intermediate overall ratings around 2.0–2.5. In general, compounds earned high overall potential only when they performed well across all or most individual ADMET criteria. Any single critically low parameter (especially a toxicity or excretion issue) tends to drag the overall score down into the moderate range or lower. This trend emphasizes that a balanced ADMET profile is required for a compound to be considered an attractive drug candidate.

Several compounds stand out for their consistently strong ADMET profiles. Most notably, compound **5** earned top marks across all five categories, making it the standout candidate. This compound combines excellent oral absorption and wide distribution with efficient metabolism/excretion and a clean safety profile, yielding an overall potential of 3 (indicating a highly favorable ADMET projection). Compound **2** also ranks among the top performers, with most criteria rated as good. Compound **2** shows excellent absorption (reflected by a high absorption score and noted high oral bioavailability) and good distribution (including moderate CNS penetration), alongside balanced metabolic clearance and no significant toxicity flags. Its overall potential is likewise in the upper range (essentially on par with compound **5**).

Compound **13** is another high-ranking candidate, distinguished especially by its outstanding absorption. It was predicted to have ~100% oral absorption (score 3), indicating it would readily reach systemic circulation. This compound’s distribution and clearance are more moderate (scores of 2 for those categories), but importantly it has a low overall toxicity risk (toxicity score 3). As a result, compound **13** still attains a high overall potential rating.

In summary, our analyses suggest compounds **5**, **2**, and **13** emerge as the top three candidates, each displaying a well-balanced ADMET profile with no major weaknesses. Their rows in the heatmap are uniformly dark or intermediate in color, reflecting generally favorable properties across the board. These compounds would be considered prime lead candidates for further development based on their composite ADMET performance.

### 2.4. Immunomodulatory Properties of Phormidesmis molle PACC 8140 Extract

**Figure 4:**
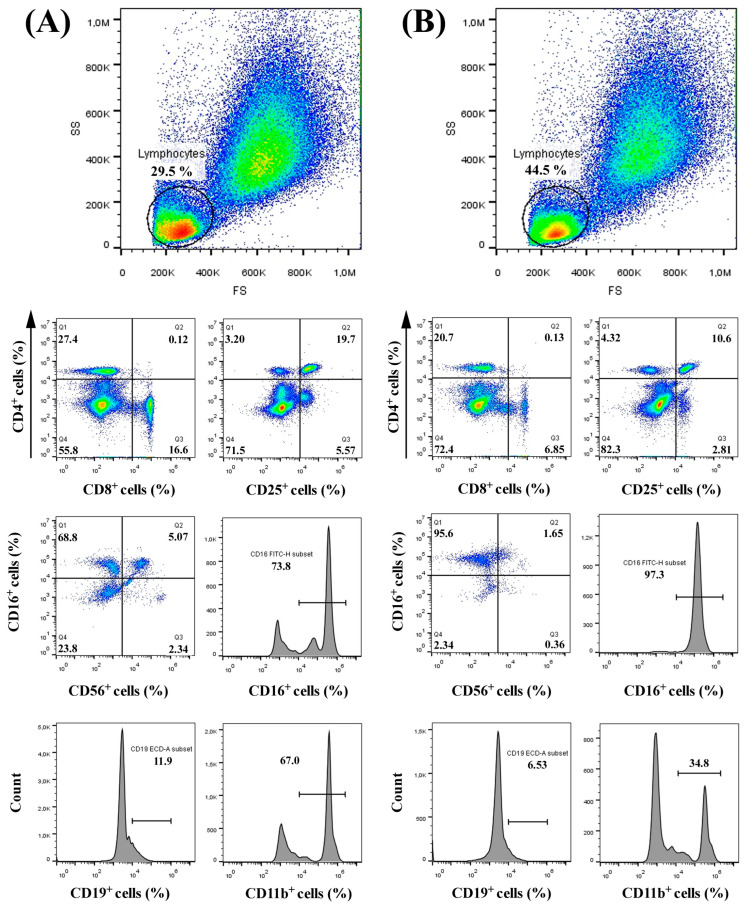
Flow cytometric immunophenotyping of human PBMCs, comparing non-treated cells (**A**) vs. cells treated for 48 h with 100 µg/mL *Phormidesmis molle* extract (**B**). (**Top panels**): Forward scatter (FSC) vs. side scatter (SSC) dot plots were used to gate the lymphocyte population. (**Lower panels**): Immunophenotyping of gated lymphocytes with markers for T cells, B cells, NK cells, and monocytes is shown using dot plots and histograms.

The results of comparative immunophenotyping of non-treated and *Phormidesmis* extract-treated human PBMCs are presented in Table 2 and Figure 4.

#### 2.4.1. Lymphocyte and Monocyte Proportions

In non-treated PBMCs (Figure 4A), the forward/side scatter gating identified lymphocytes comprising 29.5% of total events, with the remainder largely in the region of the monocytes. After 48 h exposure to 100 μg/mL *Phormidesmis* extract (Figure 4B), the lymphocyte gate expanded to 44.5% of all events. This increase in the lymphocyte fraction coincided with a marked decrease in CD11b^+^ monocytes, which dropped from 66.4 ± 0.60% in non-treated cells to 34.4 ± 0.87% after treatment (Table 2, Figure 4). Our data suggest a shift in leukocyte composition favoring lymphocytes (particularly certain subsets, detailed below) at the expense of monocytes. Monocyte gating histograms showed a loss of the large CD11b^+^ population after treatment, which is consistent with either selective cell death or downregulation of CD11b. Taken together, these results indicate that the *Phormidesmis* extract profoundly alters the PBMC subset balance, enriching the lymphocyte population while reducing the monocyte fraction.

#### 2.4.2. CD4^+^ and CD8^+^ T Lymphocyte Subsets

Performed flow cytometry analysis revealed a decrease in both CD4^+^ and CD8^+^ T cells in response to the extract treatment (Table 2, Figure 4B). In non-treated PBMCs, CD4^+^ T cells constituted 32.2 ± 4.30% of lymphocytes, whereas CD8^+^ T cells comprised 21.5 ± 4.50% (Table 2, Figure 4A). Following extract treatment, the CD4^+^ T cell proportion dropped to 21.93 ± 3.13%, and CD8^+^ T cells fell to 7.22 ± 0.41% of gated cells. The CD4:CD8 ratio was changed from 1.6 (in non-treated culture) to 3.0 (in extract-treated culture). Notably, the double-negative (CD4^−^CD8^−^) lymphocyte fraction increased substantially from 55.6 ± 2.12% in controls to 72.2 ± 2.86% after treatment, suggesting that non-T lymphocytes (or T cells lacking both markers) became dominant in the lymphocyte gate. These changes imply that the *Phormidesmis* extract causes a relative loss or redistribution of conventional T cells. The reduction in CD4^+^ and CD8^+^ frequencies could result from either direct cytotoxic effects on T cells or an expansion of other populations. Both CD4^+^ and CD8^+^ T-cell subsets were reduced after exposure to the extract.

#### 2.4.3. CD25^+^ Activated T Cells

Analysis of CD25 expression on CD4^+^ T cells revealed a decrease in activated CD4^+^CD25^+^ T regulatory cells after treatment. In the control non-treated samples, 19.1 ± 0.79% of CD4^+^ cells co-expressed CD25 (Table 2, Figure 4A), whereas only 10.9 ± 0.79% did so in the samples treated with *Phormidesmis* extract (Table 2, Figure 4B). This nearly 50% reduction in the CD4^+^CD25^+^ population indicates that the extract diminished the fraction of activated (CD25^+^) phenotype among the lymphocytes.

#### 2.4.4. NK Cells (CD56^+^ and CD16^+^ Subsets)

We found that the most dramatic immunological changes caused by the *Phormidesmis* extract was the expansion of Natural Killer (NK) cell populations (CD3 negative), identified by CD56 and CD16 markers. In non-treated PBMC, 67.7 ± 0.95% of NK cells were CD56^+^CD16^−^ and 6.07 ± 0.98% were CD56^+^CD16^+^ (Table 2, Figure 4A). After 48 h treatment with *Phormidesmis* extract, the CD56^+^CD16^−^ subset increased to 94.66 ± 0.90% while the CD56^+^CD16^+^ subset decreased to 1.85 ± 0.19% (Table 2, Figure 4B). The minor CD56^−^CD16^+^ fraction also declined (from 1.93 ± 0.46% to 0.25 ± 0.12%). Consistent with this shift, the overall CD16^+^ lymphocyte fraction increased from 73.77 ± 0.96% in the controls to 96.51 ± 0.54% in the treated samples (Table 2, Figure 4). This indicates expansion of NK cells with 22%. These findings demonstrate that *Phormidesmis* extract has a potent NK-cell stimulatory effect, driving the accumulation of atypical CD56^−^ negative NK cells. The presence of a large CD56^−^CD16^+^ population is particularly notable, as this subset is rarely seen in such high proportions under normal conditions. Overall, the extract-treated PBMCs show enhanced NK cell frequencies and altered NK phenotype, suggestive of a strong innate immune activation.

#### 2.4.5. B Cells (CD19^+^)

B lymphocyte frequency was reduced after extract exposure. In non-treated cultures, CD19^+^ B cells constituted 11.9 ± 1.70% (Table 2, Figure 4A, histogram). Following treatment, the CD19^+^ population declined to 6.06 ± 0.42% (Table 2, Figure 4B). This two-fold reduction in B cells indicates that *Phormidesmis* extract either selectively suppresses B cells or causes preferential expansion of other compartments. Given that the total lymphocyte gate increased while B cells decreased, our data suggest a dilution of B cells by expanding NK cells, and possibly direct negative effects on B cell survival. The extract-treated PBMCs had a substantially lower B cell:lymphocyte ratio than controls. No significant upregulation of CD19 or atypical B cell subsets was observed. Instead, the absolute number of CD19^+^ events fell in treated samples (as indicated by lower histogram counts).

In summary, major immune cell populations were differentially affected by the cyanobacterial extract—NK cells were enriched, whereas B cells and monocytes were diminished compared to non-treated PBMCs.

#### 2.4.6. Suppression of Th1-Type Cytokines (IFN-γ, TNF-α, IL-2) by *Phormidesmis molle* PACC 8140 Extract

**Figure 5:**
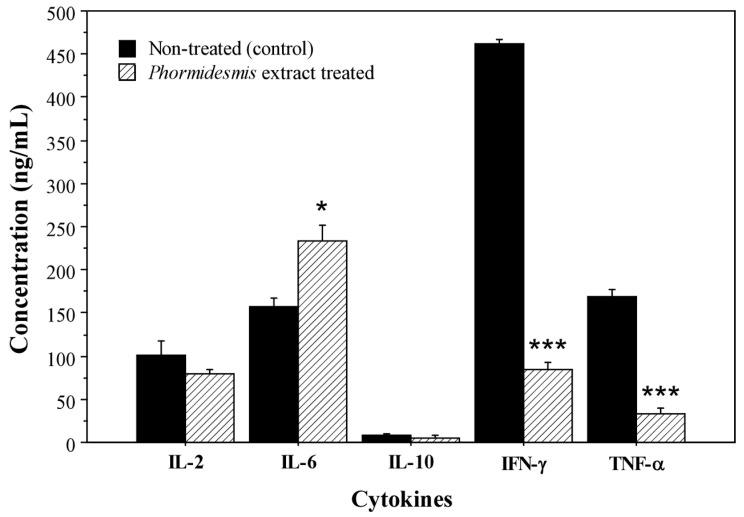
Cytokine production by cultured human PBMCs treated for 48 h with 100 µg/mL *Phormidesmis molle* extract. Supernatants were assayed by ELISA. Asterisks denote statistically significant differences from the non-treated cells (* *p* < 0.05, *** *p* < 0.001) as determined by Mann–Whitney U test. Results are presented as means ± SD (*n* = 6).

After 48 h of exposure to *Phormidesmis* extract, PBMCs showed a marked decrease in key Th1 proinflammatory cytokines compared to non-treated cells. Interferon-gamma (IFN-γ) concentrations were dramatically reduced by the extract, falling from 461 ng/mL (non-treated) to 84 ng/mL (extract-treated) (Figure 5). This represents a significant suppression of IFN-γ production, indicating that *Phormidesmis* extract strongly inhibits this T cell/NK cell cytokine. Similarly, tumor necrosis factor-alpha (TNF-α) levels were significantly lower in extract-treated cultures (32 ng/mL) versus non-treated controls (169 ng/mL) (Figure 5). The pronounced decline in TNF-α suggests that the extract attenuates monocyte/macrophage activation or viability. Interleukin-2 (IL-2), a T cell growth factor, was slightly lower in the treated PBMCs (72 ng/mL) compared to non-treated (200 ng/mL) (Figure 5). This modest decrease in IL-2 did not appear statistically significant, but it aligns with the overall trend of reduced T cell cytokine secretion upon extract treatment. Together, these results demonstrate that *Phormidesmis* extract profoundly suppresses the production of major Th1-associated cytokines IFN-γ and TNF-α, with a minor dampening effect on IL-2, compared to non-treated PBMCs.

#### 2.4.7. Increased IL-6 Production with Minimal IL-10 Response by *Phormidesmis molle* PACC 8140 Extract

In contrast to its inhibitory effects on IFN-γ and TNF-α, the *Phormidesmis* extract enhanced the secretion of interleukin-6 (IL-6) (Figure 5). IL-6 concentrations were higher in treated PBMC supernatants (234 ng/mL) than in controls (158 ng/mL) after 48 h. This increase of 48% suggests that the extract actively stimulates IL-6 release, a cytokine associated with acute inflammatory and innate immune responses. Conversely, interleukin-10 (IL-10), an anti-inflammatory cytokine, remained at very low baseline levels in both non-treated and treated cultures (Figure 5). Non-treated PBMCs produced minimal IL-10, and the *Phormidesmis* extract did not induce any notable increase in IL-10. Thus, no significant IL-10 response was elicited by the extract within 48 h. The combination of elevated IL-6 without a corresponding rise in IL-10 illustrates that *Phormidesmis* extract skews the cytokine profile toward certain innate inflammatory signals (IL-6) while failing to trigger an anti-inflammatory IL-10 feedback. This selective modulation resulted in a net cytokine profile characterized by increased IL-6 and markedly reduced IFN-γ/TNF-α, with IL-2 mildly lowered and IL-10 unchanged at baseline levels.

## 3. Discussion

### 3.1. Predominant Compound Classes and Bioactivity Potential

The chemical composition of the *Phormidesmis molle* PACC 8140 extract spans a spectrum from non-polar terpenoids and aromatics to polar lipids and sugars. The non-polar fraction contained most of the classical secondary metabolites (including terpenoid acids, chalcones, and cyclic peptides), whereas the polar fraction was enriched in primary metabolites and conjugates (sugars, sulfolipids, and osmolytes).

Geniposide, found in the non-polar/negative fraction, is traditionally isolated from plant sources and exhibits multiple pharmacological effects (anti-inflammatory, hepatoprotective, neuroprotective, etc.) [10]. Its presence here (in significantly higher abundance in the *Phormidesmis* extract than in controls) could indicate either a cyanobacterial glycoside or a biotransformed analog with similar structure.

Anabaenopeptin peptides (to which paltolide C is structurally related) are well-known cyanobacterial oligopeptides that often act as enzyme inhibitors [11]. These cyclic peptides commonly inhibit serine proteases and phosphatases, contributing to the chemical defense of the organisms or signaling [12]. Our detection of an anabaenopeptin-like peptide here is consistent with *Phormidesmis* possessing non-ribosomal peptide synthetase pathways, and it underscores the potential for bioactive protease inhibitors in the extract.

Moronic acid, detected in the non-polar/positive fraction, is a pentacyclic triterpenic acid known with antiviral properties—a derivative of moronic acid has shown potent anti-HIV activity in vitro [13] and the compound itself exhibits anti-herpes and anti-inflammatory effects [14]. The identification of moronic acid (or an isomer) in the *Phormidesmis* extract is significant, as it represents a bioactive terrestrial plant metabolite appearing in a prokaryotic source, which could indicate either a true biosynthetic occurrence or horizontal acquisition. Nootkatone (another compound found in this fraction) is a biologically active volatile. It was recently registered as a biopesticide [15] due to its ability to repel and kill ticks and mosquitoes, and it also has antifungal properties. Its presence here (absent in controls) suggests *Phormidesmis* as a producer of nootkatone or a structural analog.

SQDGs are sulfur-containing glycerolipids ubiquitous in photosynthetic organisms, including cyanobacteria [16]. Certain SQDG have demonstrated antiviral (e.g., inhibiting DNA polymerase or HIV) and antitumor activities [17]. The presence of SQDG in the polar fraction (with significantly higher abundance in the *Phormidesmis* sample vs. control) underscores the extract’s content of membrane-derived bioactive lipids. The detection of SQDG and related lipids is particularly noteworthy, as these sulfolipids not only confirm the cyanobacterial origin (being common in thylakoid membranes) but also contribute potential bioactivity (e.g., antiviral properties) [17]. Ergothioneine is also known to be synthesized by cyanobacteria and other microbes [18], and it serves as a powerful antioxidant and cytoprotective compound. Its high abundance in the *Phormidesmis* polar extract (with minimal presence in controls) suggests that this organism produces ergothioneine as part of its stress defense mechanism. This finding is significant as ergothioneine has emerging nutraceutical and therapeutic value due to its radical-scavenging and cell-protective activities [18].

The ADMET analysis of the selected 22 chemical compounds showed that most of them are soluble in water. The only exception was compound **9** (Adonirubin) with a value below −6 mol/L. This ketocarotenoid, together with adonixanthin, is considered as an alternative to the commercially available astaxanthin, which is a proven antioxidant, anticancer and antimicrobial agent [19]. Of the 22 compounds analyzed, three inhibited P-gpI but not P-gpII (compounds **8**, **13**, and **16**), while two compounds inhibited P-gpII but not P-gpI (compounds **9** and **14**). Ten of the compounds analyzed inhibited both P-gpI and P-gpII, and seven did not block P-gp. P-glycoprotein inhibitors are substances that block or reduce the activity of the P-gp transporter, a protein that helps remove drugs from cells. By inhibiting P-gp, these inhibitors can increase the concentration of drugs in cells, potentially improving their effectiveness or overcoming drug resistance. Many synthetic and natural products have been found to have inhibitory or modulating effects on drug transport proteins. The log *p* value is an important factor in the study of these inhibitors and has a crucial role on the absorption, distribution, metabolism and excretion (ADME) properties of candidate P-gp inhibitors [20].

The observed ADMET patterns provide clear guidance for in the pharmaceutical industry. Compounds like **5**, **2**, and **13**, which score well across all ADMET dimensions, should be prioritized as lead candidates. Their lack of any major liabilities implies a higher likelihood of success in preclinical models and future clinical testing, since good absorption, distribution, and safety profiles improve the chances that a drug can reach therapeutic targets at effective concentrations without dose-limiting toxicity. In practical terms, these top-tier candidates would move forward into further in vivo studies or chemical optimization with confidence in their drug-like behavior.

### 3.2. Cytotoxicity

The present cytotoxicity profile of the non-polar fraction from *Phormidesmis* extract against human colon cancer cell lines (Caco-2, HT-29, and LS-180) is consistent with earlier evidence that *P. molle* produces bioactive metabolites with antiproliferative potential, while also highlighting important differences likely driven by extraction chemistry, target cell type, and endpoint selection. Our results showed that the extract reduced viability in a concentration- and time-dependent manner, with LS-180 being the most sensitive cell line and displaying the lowest IC_50_ values (63 µg/mL), followed by Caco-2 (71 µg/mL) and HT-29 (94 µg/mL). Across the tested range (50–200 µg/mL), the strongest effects were reproducibly observed at 200 µg/mL, particularly after 72 h, supporting a biologically meaningful growth-inhibitory/cytotoxic activity of the crude extract toward colorectal carcinoma models.

The observed morphological changes after treatment with a *Phormidesmis* extract indicated pronounced cytotoxic and apoptosis-associated effects across the examined colorectal cancer cell lines. In Caco-2 cultures, many treated cells were detached from the plates. Such rounding and reduced adhesion are well-established hallmarks of apoptotic cell death [21,22], and the marked collapse of the epithelial monolayer suggests extensive loss of viability. A comparable response was found in HT-29 cells, where cell rounding, detachment from the culture surface, and formation of gaps within the monolayer were also observed, which is consistent with previously reported pro-apoptotic morphological changes [22,23]. Similarly, LS-180 cells displayed reduced adhesion and cellular shrinkage after treatment. Our findings support the conclusion that the tested *Phormidesmis* extract induces substantial apoptosis-like cell death in colorectal cancer cells, as reflected by the consistent loss of adherence, cellular rounding, and disruption of monolayer integrity across all evaluated cell lines.

A relevant example for comparison is the earlier report on two *P. molle* strains [2] demonstrating pronounced in vitro anticancer activity using multiple cancer models and complementary endpoints (MTT viability, [^3^H]-thymidine incorporation, clonogenicity, and morphology). In that study, we showed that exposure to *P. molle* extracts or growth media induced cytoskeletal and microtubule alterations with dose-dependent destruction of adherent monolayers, significant MTT-detected cytotoxicity on selected cancer lines at 24 h, and strong antiproliferative effects (inhibition of thymidine incorporation) in adherent cancer cells [2]. While our IC_50_ values fall in the 60–100 µg/mL range (higher than the concentrations highlighted previously), the directionality is concordant—*P. molle* derived preparations can suppress cancer cell growth and viability, and the magnitude of effect is plausibly contingent on strain/biomass, and solvent system. Importantly, the differential sensitivity observed here (LS-180 > Caco-2 > HT-29) may reflect distinct metabolic states and death-resistance programs among colon cancer subtypes, implying that *P. molle* extracts could exhibit context-dependent anticancer activity rather than uniform cytotoxicity across colorectal models.

In another study [1], we showed that human cell models were more sensitive than mouse cells and that certain cancer cell lines were more responsive than a normal human cell line, suggesting a potential therapeutic window (at least for some preparations) alongside a clear need for safety profiling. In this context, our moderate but consistent cytotoxicity toward colon cancer cells supports anticancer promise, but it also reinforces the requirement to evaluate selectivity against non-malignant intestinal/epithelial controls and to deconvolute the crude mixture into defined fractions.

Georgiev et al. showed that *P. molle* biomass contains polysaccharide complexes and that cyanobacterial complexes/extracts can exert in vitro growth-inhibitory effects on colorectal cancer cells, with effects linked to lysosomal activity modulation [5]. Taken together, the converging evidence indicates that *P. molle* is a credible source of anticancer-relevant bioactivity, but that its crude extracts likely comprise multiple compound classes (e.g., alkaloid-like small molecules, complex polysaccharides, and other secondary metabolites) with potentially distinct (and possibly synergistic) modes of action.

### 3.3. Immune Modulation

The exposure to *Phormidesmis* extract caused an overall loss of conventional T lymphocytes (CD4^+^ and CD8^+^). Activated (CD4^+^CD25^+^) T-cells were similarly reduced (from 19.1 ± 0.8% to 10.9 ± 0.8%) and B-cells (CD19^+^) from 11.9 ± 1.7% to 6.06 ± 0.42%.

The pronounced expansion of CD3^−^CD56^−^CD16^+^ NK cells induced by *Phormidesmis* extract is a striking immunomodulatory effect that mirrors phenomena observed in chronic viral infections. In HIV-1 infection, for example, an expanded CD56^−^CD16^+^ NK subset emerges at the expense of normal NK cells [24]. These CD56-negative NK cells are typically very scarce in healthy individuals but increase significantly under chronic immune stimulation, such as HIV or hepatitis C virus viremia [25]. Our findings are consistent with these reports. After exposure to the cyanobacterial extract, CD3^−^CD56^−^CD16^+^ cells rose from 67.7 ± 0.95% to 94.66 ± 0.90% of lymphocytes, resembling the high frequencies seen in chronic infections [26]. This suggests that the extract can acutely trigger NK cell differentiation or expansion pathways that usually develop over long-term disease. Notably, HIV-driven CD56^−^ NK cell expansion is associated with dysfunction, including low natural cytotoxicity receptor (NCR) expression and poor cytokine production [24]. Mavilio et al. reported that CD56^−^CD16^+^ NK cells from HIV patients exhibit increased inhibitory receptors, decreased activating receptors, and severely impaired cytotoxic function, contributing to overall NK cell dysfunction [27]. In our study, we did not directly assess NK cell function. However, the phenotype of the expanded cells (CD3^−^CD56^−^CD16^+^) raises questions about their activity. One possibility is that the extract-induced CD56^−^ NK subset might resemble the “dysfunctional” NK cells from chronic HIV [24]. Alternatively, these cells could be transiently activated effectors. Milush et al. found that CD56^−^CD16^+^ NK cells in HIV patients, while impaired in IFN-γ secretion, showed signs of recent activation (e.g., elevated CD107a degranulation marker and CD95) [24]. They concluded that CD56^−^ NK cells are “activated mature” NK cells with an engaged, but exhausted, phenotype during HIV-1 infection. The acute appearance of CD3^−^CD56^−^CD16^+^ NK cells in our treated PBMCs could similarly indicate a burst of NK activation followed by degranulation. In this light, the *Phormidesmis* extract appears to mimic a potent innate immune stimulus, driving NK cells into an activated state that is often only observed under chronic immunopathological conditions.

Crucially, recent research suggests that CD56^−^CD16^+^ NK cells are not uniformly inert, but may have specialized functions. Orrantia et al. demonstrated that these cells, when properly identified (using NKp80 to exclude contaminating myeloid cells), are not completely dysfunctional [25]. Their study showed CD56^−^ NK cells retain some cytotoxic capacity and can respond under certain conditions, contradicting the notion that they are purely anergic [25]. Consistent with this, Forconi et al. proposed that CD56^−^CD16^+^ NK cells represent an adaptive cytotoxic subset developed during chronic infections [28]. In endemic Burkitt lymphoma (associated with EBV and malaria), these cells accumulated and appeared to mediate antibody-dependent cellular cytotoxicity (ADCC) via Fc receptors and complement, albeit with altered signaling pathways. Forconi et al. found that CD56^−^ NK cells retain cytotoxic potential, primarily through antibody-mediated mechanisms, and are not truly exhausted despite reduced responsiveness to cytokines [28]. They suggest this subset is an unconventional effector adapted to chronic immune stimulation, possibly to contain infections while avoiding excessive inflammation [28]. Our observation that *Phormidesmis* extract rapidly generates a large CD3^−^CD56^−^CD16^+^ population might indicate the extract engages similar pathways of NK cell activation/alteration. One intriguing insight is that CD56^−^CD16^+^ NK cells can perform ADCC comparably to conventional NK cells [26]. The authors highlight that, although CD56^−^ NK cells have diminished natural cytotoxicity receptor function, their ability to kill opsonized targets via CD16 (FcγRIII) may be intact or even accentuated [26]. In our context, the extract-induced NK cells could potentially be highly competent in ADCC (through CD16 binding of antibodies), even if they resemble “dysfunctional” NK by phenotype. This aligns with the perspective that these cells are functionally reoriented rather than simply suppressed.

The cytokine profile observed after treatment with *Phormidesmis* extract reflects a distinctive pattern of immune activation. Generally, microbial products are known to engage innate immune receptors and trigger robust proinflammatory cytokine release. For example, bacterial lipopolysaccharide (LPS) from cyanobacteria can strongly activate immune cells, leading to elevated IL-6, TNF-α, and IFN-γ production [29]. Similarly, certain cyanobacterial toxins such as lyngbyatoxin potently induce a broad spectrum of cytokines in human PBMCs (including TNF-α, IL-1β, IL-6, as well as IL-10 and Th2 cytokines IL-4 and IL-5) [30]. These examples underscore that many microbial or cyanobacterial extracts function as immune stimulants, provoking an acute inflammatory response dominated by proinflammatory cytokines. Consistent with this paradigm, we found that *Phormidesmis* extract did increase IL-6, indicating activation of innate immune cells (likely monocytes) to mount an acute-phase response. However, the extract’s effects were not uniformly proinflammatory—it failed to elevate TNF-α or IFN-γ, which are typically seen in classical innate activation by endotoxins [29]. This suggests that while *Phormidesmis* components can trigger certain innate pathways (e.g., those leading to IL-6 release), they may simultaneously suppress or bypass other pathways required for TNF-α and IFN-γ induction. Thus, it is conceivable that *Phormidesmis* extracts harbor factors which activate innate cells enough to secrete IL-6, yet concurrently inhibit the signaling cascades (e.g., Toll-like receptor or co-stimulatory pathways) that normally lead to TNF-α and IFN-γ production. The net result is an atypical activation state—one that initiates certain inflammatory signals but blocks full-fledged proinflammatory cytokine release. Altogether, when compared to other microbial extracts, *Phormidesmis* triggers a partial immune activation (IL-6) that is counterbalanced by a targeted anti-inflammatory effect (silencing IFN-γ/TNF-α), without invoking the typical increase in anti-inflammatory cytokines. This unique profile adds to the growing literature on cyanobacterial products as complex immunomodulators rather than pure immune activators or suppressors.

## 4. Materials and Methods

### 4.1. Cyanobacterial Cultivation and Extract Preparation

*Phormidesmis molle* strain PACC 8140 (Cyanobacteriota) was obtained from the Plovdiv Algal Culture Collection (PACC) at Paisii Hilendarski University of Plovdiv, Bulgaria. This strain was chosen because it is a well-characterized laboratory strain available in our collection (PACC), its cultivation protocols are established in our laboratory, and our preliminary screening showed that it produces bioactive extracts.

The strain was cultured under sterile conditions in five 75 cm^2^ tissue culture flasks (TPP, Trasadingen, Switzerland) containing alkaline Z medium [1]. Cultures were maintained on a 12:12 h light:dark photoperiod with illumination of approximately 10 μmol photons m^−2^ s^−1^, provided by 40 W cool-white fluorescent lamps. After sufficient growth, the cyanobacterial biomass was collected by centrifugation at 4000 rpm for 15 min. The resulting cell pellet was frozen and subsequently lyophilized to obtain dry biomass for extraction. Lyophilized (freeze-dried) biomass (500 mg) was extracted using a sequential methanol–chloroform solvent protocol. First, the biomass was mixed with 3 mL of methanol and vortexed vigorously for 1 min. The suspension was then placed in an ultrasonic bath (Branson 5510R-DTH, Wilmington, NC, USA) for 20 min, with intermittent vortex mixing to enhance extraction of intracellular compounds. After sonication, 6 mL of chloroform were added to the mixture, and the sample was gently agitated on a rotary shaker (15 rpm) for an additional 20 min. Next, 3 mL of Milli-Q water were added to the mixture and vortexed for 1 min, then the preparation was centrifuged at 4000 rpm for 20 min to separate the phases. The extraction yielded a polar methanol–water phase and a non-polar methanol–chloroform phase, which were carefully separated. Each fraction was passed through a 0.2 μm hydrophobic PTFE syringe filter (Millex-FG, Merck KGaA, Darmstadt, Germany) to remove any remaining particulates.

Since our previous studies [1,2,5] showed that *Phormidesmis molle* extracts have pronounced cytotoxic and anticancer properties against both adhesive and suspension cancer cell lines, in order to identify *Phormidesmis molle*-specific compounds, we used for comparison (a kind of irrelevant control) another cyanobacterial extract (from *Phormidium uncinatum* PACC 8693), which did not show such activities in our cytotoxicity tests. The extract from *Phormidium uncinatum* PACC 8693 was prepared and analyzed by LC–MS in exactly the same way as that from *Phormidesmis molle* strain PACC 8140.

For LC–MS analysis, 2 mL aliquots of each filtered fraction (non-polar and polar) were transferred into standard autosampler vials, which were then capped and placed in a Peltier-cooled autosampler tray maintained at 4 °C.

For the in vitro experiments, the solvent in each filtered *Phormidesmis molle* extract was evaporated to dryness under vacuum at 37 °C using a Savant SpeedVac concentrator (Savant Instruments Inc., Farmingdale, NY, USA). The resulting dried extracts were re-dissolved in a 1:1 (*v*/*v*) mixture of DMSO and deionized water to obtain stock solutions at 5 mg/mL (*w*/*v*). For in vitro assays, these stock solutions were further diluted with RPMI-1640 Medium (Merck KGaA, Darmstadt, Germany) or Dulbecco’s phosphate-buffered saline (DPBS) (Gibco, Life Technologies, Paisley, UK) to ensure a final DMSO concentration of less than 1% (*v*/*v*) in all working solutions.

### 4.2. LC-MS Analysis and Data Treatment

The LC-MS analysis was performed by using instrumentation and procedures as previously described [7]. Briefly, the electrospray ionization (H-ESI) source was operated at a vaporizer temperature of 250 °C, with spray voltages of 4 kV in positive mode and 3 kV in negative mode. The ion transfer tube was maintained at 350 °C for positive mode and 320 °C for negative mode. Sheath gas pressure was set to 55 psi and auxiliary gas flow to 10 arbitrary units. Data acquisition was carried out in both positive and negative modes using a data-dependent Top 5 MS/MS method. Full-scan MS spectra were acquired at a resolving power of 70,000 (FWHM at *m*/*z* 200) over a mass range of 65–1100 *m*/*z* for polar compounds (HILIC and reversed-phase runs) and 134–2000 *m*/*z* for non-polar compounds. The automatic gain control (AGC) target was 1 × 10^6^ with a maximum injection time of 120 ms for full MS scans. For MS/MS scans, we used a resolution of 17,500 (FWHM) with a precursor isolation window of 1.6 *m*/*z* and a maximum ion injection time of 60 ms. A stepped higher-energy collisional dissociation (HCD) scheme was applied with a normalized collision energy (NCE) of 35% (13 eV) and a 50% energy step, resulting in an effective collision energy range of 17–53% NCE. An underfill ratio of 0.2% and a dynamic exclusion period of 10 s were enabled to reduce repeat fragmentations of the same ions. The mass spectrometer was calibrated every 24 h with the manufacturer’s standard calibration solution and tuned accordingly, and the ambient temperature was maintained at 22–26 °C during operation.

Following data acquisition, the LC–HRMS data were processed using Compound Discoverer™ Software 3.5 (Thermo Fisher Scientific, Waltham, MA, USA) with an untargeted workflow. The raw data underwent retention-time alignment, peak picking, and normalization to create a matrix of features, after which accurate masses and molecular formulas were assigned to the detected features. Related ion species (including adducts and any corresponding MS/MS fragments) were grouped into putative compound entities sharing a common molecular mass and formula. Based on these grouped features, an online search against metabolomic databases (e.g., mzCloud™, Chemspider™ and other similar resources) was performed to propose candidate identities, considering only entries from natural products databases. The resulting data matrix was used for multivariate analysis where applicable and was exported for further statistical evaluation. All features (each defined by a specific *m*/*z* and retention time) were exported to Excel and further processed with Perseus software (https://maxquant.net/perseus/, accessed on 19 October 2025) for statistical analysis [31]. Features showing significant changes (according to fold-change and *p*-value thresholds) were prioritized—their extracted ion chromatograms were inspected, and if MS/MS data were available, the fragmentation spectra were analyzed to propose tentative structures using Compound Discoverer 3.5 (https://www.thermofisher.com/order/catalog/product/COMPOUNDDISC3?SID=srch-srp-COMPOUNDDISC3, accessed on 19 October 2025), SIRIUS 5 (https://boecker-lab.github.io/docs.sirius.github.io/, accessed on 22 October 2025), and MS-Finder (https://systemsomicslab.github.io/compms/msfinder/main.html, accessed on 22 October 2025). All generated MS features were reviewed manually wherever possible, alongside the automated processing and statistical analyses. A limited number of metabolites were manually validated by comparing their fragmentation patterns with reference spectra—using either experimental or in silico MS/MS data from the METLIN (https://ngdc.cncb.ac.cn/, accessed on 22 October 2025) and mzCloud (https://www.mzcloud.org/, accessed on 22 October 2025) databases—when such data were available. Any additional manual data processing outside of Compound Discoverer was performed using Xcalibur™ 4.3 software (Thermo Fisher Scientific, Hemel Hempstead, UK; https://www.thermofisher.com/order/catalog/product/OPTON-30965, accessed on 8 November 2025) and Microsoft Excel. For quantitative comparisons, metabolite peak areas were integrated from the extracted ion chromatograms and normalized to an internal standard. Testosterone was used as the internal standard for analyses in the positive ionization mode.

### 4.3. Cell Lines and In Vitro Cytotoxicity Assays

Our previous studies on the biological activity of *P. molle* extracts were mainly focused on polar extracts [1,2,5] with an emphasis on cyanotoxins [1], polysaccharides [5] or other compounds with toxic and possible selective action on various cancer cell lines [2]. In the present study, we decided to complement the previous results with data on the cytotoxicity of non-polar fractions of *P. molle* on three colon cancer cell lines (Caco-2, HT-29 and LS-180), for which no data were available so far. In addition, our studies on non-polar fractions from other cyanobacterial species showed the presence of interesting compounds and biological activity [32].

Human colorectal adenocarcinoma cell lines Caco-2 (ATCC HTB-37™), HT-29 (ATCC HTB-38™), and LS-180 (ATCC CL-187™) were used. Cells were routinely maintained in Dulbecco’s Modified Eagle Medium (DMEM, high glucose) supplemented with 10% heat-inactivated fetal bovine serum (FBS), 100 U/mL penicillin and 100 μg/mL streptomycin (complete DMEM) (all from Merck KGaA, Darmstadt, Germany). Cultures were grown at 37 °C in a humidified incubator with 5% CO_2_. Cells were expanded in 75 cm^2^ flasks (TPP, Trasadingen, Switzerland) to 80–90% confluence, then washed with PBS and detached using trypsin-EDTA. Viability was confirmed by trypan blue exclusion test before seeding. The resulting cell suspension was adjusted to the required concentration (4 × 10^4^ cells/well) for plating. Cells were plated into 96-well flat-bottom plates (TPP, Trasadingen, Switzerland) and allowed to attach for 24 h. After the 24 h attachment period, the growth medium was replaced with fresh complete DMEM containing the *Phormidesmis molle* extract at the desired concentrations (50, 100, 200 μg/mL). Control wells received the same volume of vehicle (DMSO) without extract. Cells were then incubated for an additional 24, 48 or 72 h under standard culture conditions (37 °C, 5% CO_2_). At the end of the treatment period, 3-(4,5-dimethylthiazol-2-yl)-2,5-diphenyltetrazolium bromide (MTT, Merck KGaA, Darmstadt, Germany) was added to each well to a final concentration of 0.5 mg/mL. Plates were incubated for 3 h at 37 °C to allow viable cells to reduce MTT to insoluble formazan. After incubation, the medium was carefully removed and 100 μL of DMSO was added to each well to dissolve the formazan crystals. Plates were gently shaken for 10 min to ensure complete solubilization. Absorbance was measured at 570 nm using a SpectraMax i3x instrument (Molecular devices, San Jose, CA, USA). Cell viability assays were conducted in triplicates for each condition and presented as the percentage of the vehicle-treated control wells. At the end of the 72 h experimental period, and prior to MTT addition, microscopic images of both untreated cells and cells exposed to the extract at the highest tested concentration (200 µg/mL) were captured to evaluate potential morphological alterations. Imaging was performed using an Inverso inverted light microscope (Medline Scientific, Chalgrove, Oxfordshire, UK) with a Si-3000 digital camera and accompanying software (Medline Scientific, Chalgrove, Oxfordshire, UK).

IC_50_ values (concentration causing 50% reduction in viability) were calculated from concentration–response curves using a four-parameter logistic (4PL) nonlinear regression model (Hill equation). Cell viability data, expressed as percentage of non-treated control, obtained at 24, 48, and 72 h were pooled and analyzed as a function of extract concentration. The IC_50_ was defined as the concentration producing 50% inhibition between the upper and lower asymptotes of the fitted curve. Nonlinear regression analysis was performed using GraphPad Prism software (version X, GraphPad Software, San Diego, CA, USA), following established guidelines for accurate IC_50_ estimation [33,34].

### 4.4. ADMET Prediction

From the identified putative compounds in the individual fractions of *P. molle*, based on the peak area and the ratio of the peak area of the respective compound found in the *P. molle* extract and in the *Phormidium uncinatum* extract (control extract) (Supplementary Materials, Chemical_composition.xlsx), as well as on the insufficient study of the biological activity of these compounds, we have selected 22 compounds for next analyses. The selection was made in this way, because the highest peak area indicates the most abundant compound among the identified ones and it is likely that these compounds are responsible for the demonstrated biological activity compared to the lower or lack of such in the control *P. uncinatum* extract. Selected 22 compounds were subjected to ADMET analysis to check whether they are suitable for development of future drugs.

In silico ADMET profiling of the selected 22 compounds identified in the *Phormidesmis molle* extract was performed using the pkCSM web server (https://biosig.lab.uq.edu.au/pkcsm/prediction, accessed on 18 November 2025). pkCSM uses graph-based structural signatures to build predictive models of pharmacokinetic and toxicity properties [9]. The chemical structures of compounds **1**–**22** (provided as SMILES strings) were submitted to the pkCSM server under default settings. This approach enabled automated prediction of key drug-like properties for the compounds found in the *Phormidesmis molle* extract.

For each compound, the pkCSM predictions included parameters from all five ADMET categories: absorption (e.g., human intestinal absorption, Caco-2 permeability), distribution (e.g., steady-state volume of distribution, blood–brain barrier penetration), metabolism (e.g., interactions with major cytochrome P450 isoforms such as CYP3A4 and CYP2D6), excretion (e.g., total clearance), and toxicity (e.g., AMES mutagenicity, hERG inhibition). In total, pkCSM provides 30 predictive outputs spanning these five ADMET categories [9]. The predicted values were assembled to generate an ADMET profile for each compound, allowing a preliminary assessment of pharmacokinetic viability and potential liabilities (such as poor absorption or toxicity risks) of the major *P. molle* compounds.

### 4.5. Immunophenotyping of Human PBMCs and Evaluation of Cytokine Levels

Three healthy volunteers (males; ages 38, 41, and 45) were enrolled in the study. All participants were non-smokers and none had undergone any medical treatment in the two months preceding blood collection. Peripheral venous blood was drawn from the cubital vein into BD Vacutainer^®^ K2EDTA tubes (Becton, Dickinson and Company, Oakville, ON, Canada) at a clinical laboratory. The basic blood parameters of all donors were within normal ranges. Written informed consent was obtained from all participants before the study commenced. The study was approved by the Local Ethics Committee of Paisii Hilendarski University of Plovdiv, Bulgaria, and conducted in accordance with the Declaration of Helsinki.

Blood samples were carefully layered onto Histopaque^®^-1077 (Merck KGaA, Darmstadt, Germany) in a sterile 15 mL conical tube. The samples were centrifuged under standard density-gradient conditions (400× *g* for 30 min at room temperature). After centrifugation, the opaque layer of peripheral blood mononuclear cells (PBMCs) at the plasma–Histopaque interface was gently aspirated and transferred into a clean conical centrifuge tube. Mononuclear cells were washed twice with sterile Dulbecco’s phosphate-buffered saline (D-PBS; Gibco^®^, Life Technologies™, Paisley, UK).

PBMCs from the three volunteers were resuspended separately in RPMI-1640 Medium supplemented with 10% heat-inactivated fetal bovine serum (FBS) and an antibiotic–antimycotic solution (all from Merck KGaA, Darmstadt, Germany). This medium was designated as complete medium. The PBMCs were then seeded into 12-well plates (TPP, Trasadingen, Switzerland) at a density of 1 × 10^6^ cells/mL. For the treatment group, 20 μL of a 5 mg/mL *Phormidesmis molle* extract solution was added to each well, resulting in a final extract concentration of 100 μg/mL in 1 mL of complete medium per well. Cells were incubated under these conditions for 48 h. Control wells contained cells incubated in complete medium with the same volume of solvent (20 μL of a 1:1 DMSO:water solution) but without *Phormidesmis* extract. All cultures were maintained at 37 °C in a humidified incubator with 5% CO_2_ in air.

Following the 48 h incubation, both treated and control cells were centrifuged at 1500× *g* for 10 min. The cell pellets were resuspended in FACS buffer consisting of D-PBS with 5% FBS and 0.05% sodium azide. Cells were stained at 4 °C for 20 min with fluorochrome-conjugated antibodies in three panels: panel I (anti-CD3, anti-CD4, anti-CD8, anti-CD25), panel II (anti-CD19, anti-CD11b), and panel III (anti-CD3, anti-CD56, anti-CD16) (all antibodies from BioLegend^®^, San Diego, CA, USA). After staining, cells were washed twice with FACS buffer and resuspended in 300 μL of FACS buffer. Immunophenotyping was performed by flow cytometry using a Cytomics FC500 instrument (Beckman Coulter Inc., Indianapolis, IN, USA).

The concentrations of cytokines IL-2, IL-6, IL-10, IFN-γ, and TNF-α in the culture supernatants were measured using human ELISA kits (LEGEND MAX^TM^; BioLegend Inc., San Diego, CA, USA) according to the manufacturer’s instructions. The plates were read at 570 nm by using the SpectraMax i3x instrument (Molecular devices, San Jose, CA, USA).

### 4.6. Statistics

Comparisons between extract treated and control groups were conducted using the Fisher’s exact test with mid-*p* correction or nonparametric Mann–Whitney U test. Statistical analyses were run with IBM SPSS Statistics (version 28.0, Armonk, NY, USA) or StatView software (version 5.0, SAS Institute, Cary, NC, USA), and *p*-values below 0.05 were considered to indicate statistical significance. Data are reported as the mean ± standard deviation (SD) of six replicates for the cytokines and triplicates for the cytotoxicity and flow cytometry.

## 5. Limitations and Future Directions

From a translational standpoint, the IC_50_ values obtained in colorectal adenocarcinoma cell lines indicate that the *Phormidesmis molle* extract should be regarded as a moderately active crude preparation, best suited for activity-guided fractionation rather than direct therapeutic extrapolation. A major limitation of the present study is the absence of comparative data from non-tumor colon epithelial cells, which is necessary to establish real anticancer selectivity within a relevant tissue context.

The cytotoxic assessment relied primarily on MTT-based metabolic activity, which do not distinguish between cytostatic and cytotoxic effects. Future studies should therefore incorporate complementary assays, including clonogenic survival and proliferation, to better define the nature and reversibility of growth inhibition. Mechanistic validation is also warranted, particularly in view of earlier reports describing cytoskeletal disruption and lysosome-associated effects linked to *P. molle* derived preparations. Follow-up analyses should include apoptosis and necrosis markers, cell-cycle profiling, lysosomal integrity assays, and high-resolution imaging of cytoskeletal organization.

In addition, the pronounced expansion of NK-cell populations observed in PBMC cultures requires functional validation. Assessments of NK cytotoxic activity, degranulation capacity, and cytokine production will be essential to determine whether this phenotype reflects effective immune activation or a dysregulated response.

Finally, integration of bioassay-guided fractionation with existing chemical profiling and in silico ADMET analyses represents the next step toward identifying the compounds or combinations responsible for the observed bioactivities. These approaches will clarify whether the colon cancer-directed cytotoxicity reported here can be translated into selective anticancer efficacy with an acceptable safety margin.

## 6. Conclusions

In summary, we analyzed *Phormidesmis molle* PACC 8140 extract, and several identified compounds fall into classes with known biological activity—pentacyclic triterpenes like moronic acid (antiviral and anti-inflammatory action), iridoid glycosides like geniposide (neuroprotective, anti-inflammatory action), sesquiterpenes like nootkatone (insecticidal repellent), sulfolipids like SQDG (antiviral and antitumor potential), cyclic peptides like anabaenopeptins (protease inhibitors), and thiol antioxidants like ergothioneine (cytoprotective action). The dominance of these classes suggests that the *Phormidesmis* extract is a rich source of compounds that could underlie the observed bioactivities (e.g., antimicrobial or cytotoxic effects) of cyanobacterial extracts. The combination of LC-MS/MS profiling and database matching has thus allowed us to catalog the putative chemical constituents of each fraction and highlight those compound classes most likely to contribute to the biological efficacy of the extract. Such extensive metabolite profiling paves the way for targeted isolation and detailed investigation of bioactive compounds derived from the cyanobacterial extract.

ADMET analysis performed on 22 selected compounds identified compounds **2**, **5**, and **13** as the most balanced and favorable candidates for pharmaceutical development, combining strong bioavailability with acceptable metabolism and low toxicity. Compound **16** remains a candidate of interest but requires further genotoxicity evaluation, while compound **19** demonstrates promise contingent upon the resolution of its metabolic liabilities.

The *Phormidesmis* extract markedly reduced colon cancer cell viability in a dose- and time-dependent manner, indicating promising anticancer activity. Morphological changes were associated with cell rounding, shrinkage, and detachment from the plate surface, disrupting the monolayer. These characteristics correspond to the classic hallmarks of apoptosis. In addition, it exerts a rapid and striking reshaping of the immune cell landscape, mirroring several hallmarks of chronic viral infections while operating within an acute timeframe. Key findings include the expansion of CD3^−^CD56^−^CD16^+^ NK cells—an unconventional subset associated with chronic immune activation—and the concurrent depletion of both T and B lymphocytes. Such a profile suggests that the extract mimics certain aspects of prolonged antigenic stimulation or cytokine therapy, yet does so swiftly and without ongoing infection. The potential to trigger profound NK cell responses via natural compounds opens exciting therapeutic possibilities, especially for enhancing innate immunity in infectious or oncological contexts. Moreover, the observed cytokine milieu (particularly elevated IL-6 in the absence of classic Th1 cytokines) implies a shift toward antibody-mediated or acute-phase responses rather than T cell-driven immunity. This could reduce tissue-damaging inflammation while still offering early defense.

## Figures and Tables

**Table 1:** Selected compounds for ADMET profiling.

Number	Name of the Selected Compounds
**1**	1,3,5-Tris[4-hydroxy-3,5-bis(2-methyl-2-propanyl)benzyl]-1,3,5-triazinane-2,4,6-trione
**2**	Nootkatone
**3**	1-(1-Benzyl-4-piperidinyl)-1-({4′-[(isobutylamino)methyl]-4-biphenylyl}methyl)-3-phenylurea
**4**	1-{3-[(2R,3S,4R,5R,8R,10R,11R,12S,13S,14R)-13-[(2,6-Dideoxy-3-C-methyl-3-O-methyl-α-L-ribo-hexopyranosyl)oxy]-2-ethyl-3,4,10-trihydroxy-3,5,8,10,12,14-hexamethyl-15-oxo-11-{[3,4,6-trideoxy-3-(dime thylamino)-β-D-xylo-hexopyranosyl]oxy}-1-oxa-6-azacyclopentadecan-6-yl]propyl}-3-(2-naphthyl)urea
**5**	(3E)-8-Phenyl-3-octen-2-one
**6**	(2S,4S,5S)-6-Cyclohexyl-4-hydroxy-2-isopropyl-5-{[(2S)-2-({(2S)-1-[4-(methoxymethoxy)-1-piperidinyl]-1-oxo-3-phenyl-2-propanyl}oxy)hexanoyl]amino}-N-[4-(4-morpholinyl)butyl]hexanamide
**7**	1{3[(2R,3S,4R,5R,8R,10R,11R,12S,13S,14R)-13-[(2,6-Dideoxy-3-C-methyl-3-O-methyl-α-L-ribo-hexopyranosyl)oxy]-2-ethyl-3,4,10-trihydroxy-3,5,8,10,12,14-hexamethyl-15-oxo-11-{[3,4,6-trideoxy-3-(dime thylamino)-β-D-xylo-hexopyranosyl]oxy}-1-oxa-6-azacyclopentadecan-6-yl]propyl}-3-(2-naphthyl)urea
**8**	2,6-Bis(4-ethylphenyl)perhydro-1,3,5,7-tetraoxanaphth-4-ylethane-1,2-diol
**9**	Adonirubin
**10**	(2S)-1-(β-D-Galactopyranosyloxy)-3-[(9E)-9-hexadecenoyloxy]-2-propanyl (9E,12E)-9,12-octadecadienoate
**11**	1-(1-Benzyl-4-piperidinyl)-1-({4′-[(isobutylamino)methyl]-4-biphenylyl}methyl)-3-phenylurea
**12**	N~1~,N~4~-Bis(4-{[6-methoxy-2-(2-methyl-2-propanyl)-8-quinolinyl]amino}pentyl)-L-aspartamide
**13**	(5β,15β)-3,16-Dioxo-14,15-epoxybufa-20,22-dienolide
**14**	1,3-Bis(tetradecanoyloxy)-2-propanyl (5Z,8Z,11Z,14Z)-5,8,11,14-icosatetraenoate
**15**	(3β)-3-({1-[4-(Cyanomethyl)phenyl]-1H-1,2,3-triazol-4-yl}methoxy)lup-20(29)-en-28-oic acid
**16**	(4R,8S,9R,11R)-11-Methyl-2,7-bis(methylene)-6,12-dioxo-5,14-dioxatricyclo[9.2.1.0~4,8~]tetradec-1(13)-en-9-yl methacrylate
**17**	1-Allyl-2-phenylpiperazine
**18**	(2S)-2-[[1-(5-hydroxypentyl)indazole-3-carbonyl]amino]-3-methylbutanoic acid
**19**	Aceperone
**20**	Laurylsulfonic acid
**21**	Methyl 4-(24-methoxy-7,12,24-trioxochol-3-en-3-yl)-3,7,12-trioxocholan-24-oate
**22**	Paltolide C

**Table 2:** Changes in PBMC immune cell subpopulations after exposure to the *Phormidesmis* extract.

CD Markers	Non-Treated PBMCs (%)			Mean ± SD (%)	Extract-Treated PBMCs (%)			Mean ± SD (%)	*p*-Values *
	Sample 1	Sample 2	Sample 3		Sample 1	Sample 2	Sample 3		
CD3^+^	65.7	67.5	63.0	65.4 ± 2.26	56.3	59.3	53.7	56.43 ± 2.80	*p* = 0.024
CD4^+^	27.4	35.7	33.5	32.2 ± 4.30	20.7	25.5	19.6	21.93 ± 3.13	*p* = 0.008
CD8^+^	16.6	22.8	25.35	21.5 ± 4.50	6.85	7.16	7.67	7.22 ± 0.41	*p* < 0.001
CD4^+^CD25^+^	19.7	18.2	19.4	19.1 ± 0.79	10.6	10.3	11.8	10.9 ± 0.79	*p* = 0.008
CD19^+^	11.9	10.3	13.7	11.9 ± 1.70	6.53	5.97	5.7	6.06 ± 0.42	*p* = 0.023
CD11b^+^	67.0	66.4	65.8	66.4 ± 0.60	34.8	33.4	35.0	34.4 ± 0.87	*p* < 0.001
CD3^−^CD56^+^CD16^+^	5.07	6.12	7.02	6.07 ± 0.98	1.65	1.88	2.04	1.85 ± 0.19	*p* = 0.020
CD3^−^CD56^+^CD16^−^	2.34	1.44	2.02	1.93 ± 0.46	0.36	0.12	0.28	0.25 ± 0.12	*p* = 0.061
CD3^−^CD56^−^CD16^+^	68.8	67.4	67.0	67.7 ± 0.95	95.6	93.8	94.6	94.66 ± 0.90	*p* < 0.001

* Due to the small number of samples studied (*n* = 3), Fisher’s exact test with mid-*p* correction was used for statistical analysis.

## Data Availability

The original contributions presented in this study are included in the article/Supplementary Material.

## Supplementary Materials

The following supporting information can be downloaded at: https://www.mdpi.com/article/10.3390/ijms27052236/s1.

## Abbreviations

The following abbreviations are used in this manuscript:

ADMET	Absorption, distribution, metabolism, excretion and toxicity
ATCC	American Type Culture Collection
BBB	Blood–brain barrier
CD	Cluster of differentiation
CNS	Central nervous system
COX	Cyclooxygenase
C-PE	C-phycoerythrin
CYP	Cytochrome P450
DGDG	Digalactosyldiacylglycerol
DMSO	Dimethyl sulfoxide
DPBS	Dulbecco’s Phosphate-Buffered Saline
ELISA	Enzyme-linked immunosorbent assay
EPS	Exopolysaccharides
ESI	Electrospray ionization
FBS	Fetal bovine serum
FcγRIII	Fc gamma receptor III
Fu	Fraction unbound
HCD	Higher-energy collisional dissociation
IC_50_	Half-maximal inhibitory concentration
IFN-γ	Interferon gamma
IL	Interleukin
LC-ESI-MS/MS	Liquid chromatography—electrospray ionization—tandem mass spectrometry
LPS	Lipopolysaccharides
MGDG	Monogalactosyldiacylglycerol
MTT	3-(4,5-dimethylthiazol-2-yl)-2,5-diphenyl tetrazolium bromide
NCE	Normalized collision energy
NK	Natural killer
PACC	Plovdiv Algal Culture Collection
PBMCs	Peripheral blood mononuclear cells
P-gp	*P*-glycoprotein
SQDGs	Sulfoquinovosyl diacylglycerols
TNF-α	Tumor necrosis factor alpha
