# Supplementary material for: Cytotoxic and Immunomodulatory Effects of Phormidesmis molle Extract on Human Cells In Vitro
Source: Int J Mol Sci. 2026 Feb 27;27(5):2236. doi: 10.3390/ijms27052236 (PMC12984838; doi:10.3390/ijms27052236)
Supplement: Supplementary file 1 [file ijms-27-02236-s001.zip › Tables-ADMET.pdf]

Table S1. Prediction of the absorption and distribution properties of selected compounds identified in the extract of *Phormidesmis molle* strain PACC 8140 with the best molecular interaction energies.

Number of the selected compound	Absorption						Distribution			
	Water solubility (log mol/L)	Caco2 permeability (log Papp in 10^{-6} cm/s)	Intestinal absorbtion (human) %	Skin permeability (log Kp)	P-glycoprotein I Inhibitor	P-glycoprotein II Inhibitor	VDss (human) (log L/kg)	Fraction unbound (human) (Fu)	BBB permeability (log BB)	CNS permeability (log PS)
1	-2.92	-0.171	91.079	-2.735	Yes	Yes	-1.073	0.352	-0.848	-1.702
2	-4.586	1.305	95.635	-1.863	No	No	0.391	0.221	0.635	-1.986
3	-3.822	1.152	86.115	-2.735	Yes	Yes	0.623	0.067	0.258	-0.19
4	-3.283	-0.295	46.835	-2.735	Yes	Yes	0.462	0.246	-2.293	-3.837
5	-4.181	1.387	96.225	-1.762	No	No	0.462	0.112	0.659	-1.495
6	-3.999	0.314	59.755	-2.732	Yes	Yes	-0.997	0.198	-1.445	-3.606
7	-3.283	-0.295	46.835	-2.735	Yes	Yes	0.462	0.246	-2.293	-3.837
8	-5.047	1.193	93.429	-2.775	Yes	No	-0.383	0	-0.175	-3.041
9	-6.844	1.249	93.266	-2.74	No	Yes	-0.411	0	-0.506	-1.129
10	-3.223	-0.27	59.213	-2.735	Yes	Yes	-1.852	0.222	-2.184	-3.485
11	-3.822	1.152	86.115	-2.735	Yes	Yes	0.623	0.067	0.258	-0.19
12	-4.24	0.147	69.409	-2.735	Yes	Yes	0.037	0.165	-1.561	-2.942
13	-4.054	1.078	100	-3.074	Yes	No	0.117	0.069	-0.245	-1.992
14	-2.993	-0.212	85.002	-2.735	No	Yes	-1.202	0.285	-1.496	-2.022
15	-3.102	0.768	96.861	-2.735	No	No	-1.286	0.04	-0.647	-1.529
16	-3.603	1.3	100	-3.05	Yes	No	-0.038	0.394	-0.546	-2.911
17	-2.9	-0.148	0	-2.735	No	No	-0.596	0.457	-1.54	-3.772
18	-3.144	1.138	56.333	-2.735	No	No	-0.765	0.111	-1.225	-3.068
19	-4.867	1.354	92.778	-2.828	Yes	Yes	0.884	0	0.106	-2.275
20	-3.263	1.439	92.654	-2.735	No	No	-0.875	0.277	0.126	-2.772
21	-3.398	0.171	100	-2.734	Yes	Yes	-1.324	0	-1.097	-2.085
22	-2.892	-0.238	0	-2.735	No	No	-0.834	0.427	-1.845	-3.849

Table S2. Prediction of the metabolic and excretory properties of selected compounds found in the extract of *Phormidesmis molle* strain PACC 8140.

Number of the selected compound	Metabolism							Excretion	
	CYP2D6 Substrate	CYP3A4 Substrate	CYP1A2 Inhibitor	CYP2C19 Inhibitor	CYP2C9 Inhibitor	CYP2D6 Inhibitor	CYP3A4 Inhibitor	Total Clearance (logml/min/kg)	Renal OCT2 Substrate
1	No	Yes	No	Yes	Yes	No	No	-0.881	No
2	No	Yes	No	No	No	No	No	1.195	No
3	Yes	Yes	No	Yes	No	Yes	No	0.639	No
4	No	Yes	No	No	No	No	Yes	-0.635	No
5	No	Yes	Yes	No	No	No	No	0.262	No
6	No	Yes	No	No	No	No	No	1.037	No
7	No	Yes	No	No	No	No	Yes	-0.635	No
8	No	Yes	No	No	No	No	Yes	1.12	No
9	No	Yes	No	No	No	No	No	0.849	No
10	No	Yes	No	No	No	No	No	2.222	No
11	Yes	Yes	No	Yes	No	Yes	No	0.639	No
12	No	Yes	No	Yes	No	No	Yes	0.964	No
13	No	Yes	No	No	No	No	Yes	0.346	No
14	No	Yes	No	No	No	No	No	2.532	No
15	No	Yes	No	No	No	No	No	-0.333	No
16	No	Yes	No	No	No	No	No	1.256	No
17	No	Yes	No	No	No	No	No	-1.455	No
18	Yes	Yes	No	No	No	No	No	-0.621	No
19	No	Yes	No	No	No	Yes	Yes	1.01	Yes
20	No	No	No	No	No	No	No	1.757	No
21	No	Yes	No	No	No	No	No	-0.609	No
22	No	Yes	No	No	No	No	No	-1.337	No

Table S3. Prediction of toxicity of selected compounds found in the extract of *Phormidesmis molle* strain PACC 8140.

Number of the selected compound	Toxicity									
	AMES toxicity	Max. tolerated dose (human) (logmg/kg/day)	hERG I inhibitor	hERG II inhibitor	Oral Rat Acute Toxicity (LD50) (mol/kg)	Oral Rat Chronic Toxicity (LOAEL) (log mg/kg_bw/day	Hepato-toxicity	Skin Sensitisation	*T.pyriformis* toxicity (log ug/L)	Minnow toxicity (log mM)
1	No	0.438	No	Yes	2.537	-1.124	Yes	No	0.285	-6.737
2	No	0.07	No	No	1.603	1.19	No	Yes	1.414	0.905
3	Yes	0.391	No	Yes	2.031	3.099	Yes	No	0.285	-0.675
4	No	-0.003	No	Yes	3.029	3.015	Yes	No	0.285	9.325
5	No	0.797	No	No	1.781	1.2	No	Yes	2.059	-0.414
6	No	-0.737	No	Yes	4.164	1.62	Yes	No	0.285	1.432
7	No	-0.003	No	Yes	3.029	3.015	Yes	No	0.285	9.325
8	No	0.443	No	No	2.492	2.938	Yes	No	0.291	0.055
9	No	-0.801	No	No	2.33	2.382	No	No	0.331	-3.196
10	No	0.296	No	No	3.289	5.005	No	No	0.285	-1.325
11	Yes	0.391	No	Yes	2.031	3.099	Yes	No	0.285	-0.675
12	No	0.222	No	Yes	2.449	1.19	Yes	No	0.285	-0.363
13	No	-0.352	No	No	2.341	2.178	No	No	0.357	0.139
14	No	0.425	No	No	2.554	0.593	No	No	0.285	-11.627
15	No	0.343	No	No	2.625	1.727	Yes	No	0.285	-3.366
16	Yes	-0.122	No	No	2.624	1.543	No	No	0.337	2.581
17	No	0.532	No	No	2.486	5.753	Yes	No	0.285	10.961
18	No	-0.621	No	No	2.542	1.206	Yes	No	0.285	0.744
19	No	-0.321	No	No	2.913	1.135	No	No	0.782	0.301
20	No	-0.575	No	No	1.622	2.693	No	Yes	0.289	-0.589
21	No	0.856	No	No	2.5	0.864	No	No	0.285	-3.325
22	No	0.508	No	No	2.483	6.529	Yes	No	0.285	11.104
